# Environmental Analytical Chemistry

**DOI:** 10.3390/molecules29020450

**Published:** 2024-01-17

**Authors:** Stefan Tsakovski, Tony Venelinov

**Affiliations:** 1 Chair of Analytical Chemistry, Faculty of Chemistry and Pharmacy, Sofia University St. Kliment Ohridski, 1 J. Bourchier Blvd., 1164 Sofia, Bulgaria; stsakovski@chem.uni-sofia.bg; 2 Chair of Water Supply, Sewerage, Water and Wastewater Treatment, Faculty of Hydraulic Engineering, University of Architecture, Civil Engineering and Geodesy, 1 Hr. Smirnenski Blvd., 1046 Sofia, Bulgaria

## 1. Introduction

Environmental analytical chemistry has evolved into a well-established interdisciplinary field (analytical chemistry, pollution chemistry, chemical engineering, etc.), and it is currently in high demand. Investigations of the Earth’s resources often exceed environmental capacity, causing various problems and changes. The management of these processes requires monitoring and analysis to gain information about organic, inorganic, and radioactive pollutants in air, water, soil, and biota. The development of new or enhancement of existing analytical methods is vital to achieve such goals. Examples include acquiring representative samples, improving sample preparation, lowering the quantification limits and measurement uncertainty, implementing appropriate methods and procedures for pollution risk assessment, revealing sources and pathways of exposure, as well as trends and the spatial distribution of analysed pollutants, among others.

The Special Issue “Environmental Analytical Chemistry” was introduced on 16 August 2021 and closed on 26 November 2023, with the submission deadline being on 20 May 2023. During this time, 18 papers were submitted seeking publication in *Molecules*.

## 2. An Overview of Published Articles

The diversity of studies related to the environment was highlighted by the papers received for publication in the SI “Environmental Analytical Chemistry” in the period from 13 October 2021 to 20 June 2023. These included the somewhat traditional topics “air”, “soil”, “sediment”, “water”, and “plant”, and emerging ones such as “e-cigarettes” (“e-liquids”).

Andrea Mara et al. (contribution 1) addressed the present lack of interest in the determination of toxic elements in electronic cigarette liquids (e-liquids). The analytical challenges for such investigations are strong matrix effects, which reflect on the existence of reliable, accurate, and validated analytical methods. In their study, the team developed and validated an ICP-MS method for the quantification of 23 elements in 37 e-liquids of different flavours, including sample pre-treatment and the optimisation of the ICP-MS conditions. Luckily, the results showed that all samples exhibited a very low amount of the investigated elements with a sum of their average concentration of ca. 0.6 mg/kg. Toxic elements in tobacco and tonic flavours (the highest and the lowest concentration of elements, respectively) were always below a few tens of μg/kg and very often below the quantification limits.

In their study, Jungmin Jo et al. (contribution 2) paid attention to some derivatives of PAHs—nitro-PAHs (NPAHs) and oxy-PAHs (OPAHs). They presented a validated method for the quantification of 18 NPAH and OPAH congeners in the atmosphere using gas chromatography coupled with chemical ionisation mass spectrometry. The application of negative chemical ionisation (NCI/MS) or positive chemical ionisation tandem mass spectrometry (PCI-MS/MS) achieved high sensitivity and selectivity for the quantification of individual NPAH and OPAH congeners without sample preparations. According to the results, the contribution of individual NPAHs and OPAHs to the total concentration differed according to the regional emission characteristics.

Markers of chemical and microbiological contamination in fitness centres were investigated by Justyna Szulc and her colleagues (contribution 3). Their study aimed to assess the particulate matter, CO_2_, formaldehyde, volatile organic compound (VOC) concentration, the number and the biodiversity of microorganisms, and the presence of SARS-CoV-2 in the air, using various analytical methods. Their results showed that >99.6% of the particles are found in the PM_2.5_ fraction. Different substances in various concentrations (CO_2_, formaldehyde, 84 VOCs phenol, D-limonene, toluene, and 2-ethyl-1-hexanol), 422 genera of bacteria, 408 genera of fungi, and the SARS-CoV-2 virus were detected in the gym.

Inductively coupled plasma mass spectrometry (ICP-MS) was used by Nimelan Veerasamy et al. (contribution 4) to measure the concentration of trace and rare earth elements (REEs) in soils from Odisha, on the east coast of India. This analytical method was validated by the use of certified reference materials. The presented estimation of enrichment factor (EF) and geoaccumulation index (Igeo) showed that Cr, Mn, Fe, Co, Zn, Y, Zr, Cd, and U were significantly enriched, and Th was extremely enriched.

How soil contamination with Cr(III) and Cr(VI) in the presence of Na_2_EDTA affects *Avena sativa* L. biomass was evaluated by Edyta Boros-Lajszner et al. (contribution 5). They assessed the remediation capacity of *Avena sativa* L. based on its tolerance index, translocation factor, and chromium accumulation, and they investigated how these chromium species affect the soil enzyme activity and physicochemical properties of soil. It was shown that the negative effect of chromium decreased the biomass of *Avena sativa* L. (aboveground parts and roots). The tolerance indices (TIs) showed that Avena sativa L. tolerates Cr(III) contamination better than Cr(VI) contamination and is of little use for the phytoextraction of chromium from the soil.

Galina Yotova et al. (contribution 6) used various indicators—primary nutrients (C, N, P), acidity (pH), physical clay content, and potentially toxic elements (PTEs: Cu, Zn, Cd, Pb, Ni, Cr, As, and Hg)—combined with chemometric and geostatistical methods to assess Bulgarian soil quality. The use of principal component analysis identified the contribution of each latent factor (the mountain soil factor, the geogenic factor, the ore deposit factor, the low nutrition factor, and the mercury-specific factor) to the overall soil quality. The spatial distribution of the soil quality patterns throughout the whole Bulgarian territory was visualised via mapping and was used to outline regions where additional measures for the monitoring of the phytoavailability of PTEs were required, -

Sediment cores were used by Tony Venelinov et al. (contribution 7) to study the temporal dynamics of anthropogenic impacts on the Pchelina Reservoir. They used ^137^Cs activity to identify the layers corresponding to the 1986 Chernobyl accident and for the calculation of the average sedimentation rate. A Mann–Kendall test was used to reveal time trends in the elements’ depth profiles (Ti, Mn, Fe, Zn, Cr, Ni, Cu, Mo, Sn, Sb, Pb, Co, Cd, Ce, Tl, Bi, Gd, La, Th, and U_nat_) within the sediments in the sampling sites. The performed principal component analysis revealed two groups of chemical elements that were linked to anthropogenic impacts. The results obtained showed that the moderately contaminated, according to the Igeo, Pchelina Reservoir surface sediment samples have low ecotoxicity.

The bioavailability and fractionation of rare earth elements were assessed by Mohammed Othman Aljahdali and Abdullahi Bala Alhassan in mangrove ecosystems (contribution 8) using multi-elemental ratios, Igeo, bio-concentration factor (BCF), and the influence of sediment grain-size types. The results obtained showed BCF values of less than one for all the REEs determined, calling for the periodic monitoring of REE concentrations in mangroves to keep track of the sources of metal contamination and develop conservation and control strategies for these important ecosystems.

Błażej Kudłak et al. (contribution 9) utilised bioluminescent bacteria (Microtox assay) to monitor contaminants of emerging concern (CEC) mixtures at environmentally relevant doses and performed the first systematic study involving three sunscreen components (oxybenzone, OXYB; 4-methylbenzylidene-camphor, 4MBC and 2-ethylhexyl 4-methoxycinnamate, EMC) and three bisphenols (BPs—BPA, BPS or BPF) (contribution 9). A breast cell line and cell viability assay were used to determine the possible effect of these mixtures on human cells. The results from toxicity modelling with concentration addition and independent action approaches showed that mixtures containing any pair of three BPs (e.g., BPA + BPS, BPA + BPF and BPS + BPF), together with one sunscreen component (OXYB, 4MBC or EMC), interacted at environmentally relevant concentrations and had strong synergy or over additive effects. Mixtures containing any pair of OXYB, 4MBC, and EMC, and one BP had a strong propensity towards concentration-dependent underestimation. The UV filters (4MBC, EMC, and OXYB) were shown to be antagonistic toward each other.

Aleksander Kravos et al. (contribution 10) utilised advanced oxidation processes (AOPs) to understand the complex degradation processes of phenol, 2,4-dichlorophenol, and pentachlorophenol from a chemical and ecotoxicological point of view. They used instrumental analyses (HPLC–DAD, GC–MS, UHPLC–MS/MS, and ion chromatography) along with ecotoxicological assessment (Daphnia magna) to study the efficiency of ozonation, photocatalytic oxidation with immobilised nitrogen-doped TiO_2_ thin films, and electrooxidation on boron-doped diamond (BDD) and mixed metal oxide (MMO) anodes. Monitoring the removal of target phenols, dechlorination, transformation products, and ecotoxicological impact, our results showed that ozonation was by far the most suitable for degradation. It showed rapid detoxification, contrary to photocatalysis, which was found to be slow and accumulated aromatic by-products.

Kullapon Kesonkan (contribution 11) proposed coconut oil as a natural solvent for the green downscaling solvent extractive determination of Cu(II) using 1,5-Diphenylcarbazide (DPC). Cu(II)-DPC complexes in an aqueous solution were transferred into the coconut oil phase, inducing a colour change, which enabled image processing on a smartphone. The developed new approach of green chemical analysis was applied to water samples in the range of 0–1 mg/mL Cu(II).

A novel biomass adsorbent based on activated carbon incorporated with sulphur-based binary metal oxide layered nanoparticles (SML-AC), including sulphur (S_2_), Mn, and Sn oxide, was synthesised by Binta Hadi Jume et al. via the solvothermal method (contribution 12). The functional groups, surface morphology, and elemental composition of the newly synthesised SML-AC were studied using FTIR, FESEM, EDX, and BET, which were applied as efficient adsorbents to remove Pb^2+^, Cd^2+^, Cr^3+^, and V^5+^ from the oil-rich region. The optimal pH, dosage, and time were found to provide a satisfactory adsorption capacity based on the Langmuir and Freundlich models.

Klaudia Stando et al. (contribution 13) evaluated the uptake of 14 veterinary pharmaceuticals by parsley from soil fertilised with manure. Pharmaceutical content (enrofloxacin, tylosin, sulfamethoxazole, and doxycycline) was determined in roots and leaves using liquid chromatography coupled with tandem mass spectrometry. Additionally, a solid–liquid extraction procedure combined with solid-phase extraction was developed, providing good recoveries for leaves and roots. The results obtained showed that enrofloxacin was present at the highest concentrations, doxycycline accumulated mainly in the roots, tylosin in the leaves, and sulfamethoxazole was found in both tissues.

## 3. Conclusions

Humanity irreversibly impacts the environment by polluting its natural vital resources—air, water, and soil. Therefore, environmental protection should become humanity’s ultimate goal. A few directions should continue to be followed: (i) the development of new and the enhancement of existing analytical methods for the analysis of pollutants (organic, inorganic, and radioactive); (ii) the use of chemometric and statistical approaches for risk assessment; (iii) the determination of point and non-point pollution sources; (iv) the clarification of exposure pathways; and (v) the determination of trends and spatial distribution of analysed pollutants.

Following the initial Special Issue’s success, a new Special Issue “Environmental Analytical Chemistry II” was introduced on 31 July 2023, with the submission deadline set to 31 May 2024 (to be closed on 27 November 2024). The Guest Editors would like to invite you to contribute to this Special Issue, so that your valuable unpublished research can find a worldwide audience among readers of *Molecules*.

